# Supplementary material for: Disease burden and clinical severity of the first pandemic wave of COVID-19 in Wuhan, China
Source: Nat Commun. 2020 Oct 27;11:5411. doi: 10.1038/s41467-020-19238-2 (PMC7591855; doi:10.1038/s41467-020-19238-2)
Supplement: Supplementary file 1 — Supplementary Information [file 41467_2020_19238_MOESM1_ESM.pdf]

·**Supplementary Information**

Disease burden and clinical severity of the first pandemic wave of novel coronavirus disease 2019 in Wuhan, China

Juan Yang, PhD, Xinhua Chen, BSc, Xiaowei Deng, MSc, Zhiyuan Chen, BSc, Hui Gong, BSc, Han Yan, BSc, Qianhui Wu, BSc, Huilin Shi, BSc, Shengjie Lai, PhD, Marco Ajelli, PhD, Cecile Viboud, PhD, Prof Hongjie Yu, PhD

Supplementary file 1. Case definition 4
Supplementary file 2. Description of active screening in Wuhan 8

Table S1. Active community universal screenings of fever cases among general population in Wuhan 8

Supplementary file 3: Disease burden and clinical severity of COVID-19 in Wuhan 9

Table S2. Summary of disease burden and clinical severity of COVID-19 in baseline and sensitivity analyses 9

Supplementary file 4: Disease burden of COVID-19, influenza pandemic in 2009 and seasonal influenza 13

Table S3. Summary of disease burden of COVID-19, influenza pandemic in 2009 and seasonal influenza 13

Supplementary file 5: Summary of severity of the COVID-19 pandemic, and 1918 and 2009 influenza pandemics 15

Table S4. Severity of the 1918 and 2009 influenza pandemics 15

Fig. S1. Hospitalization-fatality risk (HFR) 16

Fig. S2. Symptomatic case-hospitalization risk (sCHR) 17

Supplementary file 6: Estimated disease burden of COVID-19 based on ILI consultations, and SARI/pneumonia hospitalizations 18

Fig. S3. Estimated disease burden of COVID-19 based on ILI consultations, and SARI/pneumonia hospitalizations between December 1, 2019 through March 31, 2020 in Wuhan, China (mean, 95%CI) 18

Supplementary file 7: Summary of disease burden estimates of COVID-19 in Canada and the US 19

Table S5. Disease burden estimates of COVID-19 in Canada and the US 19

Supplementary file 8: Reported and age-standardized rates of 2009 pandemic influenza and seasonal influenza in USA and China 22

Table S6. Reported and age-standardized rates of 2009 pandemic influenza and seasonal influenza in USA and China 22

Supplementary file 9: Adjustment for RT-PCR sensitivity 23

Table S7. Time interval from symptom onset to diagnosis in Wuhan 23

Supplementary file 10: Summary of data used to estimate the disease burden and clinical severity of COVID-19 in Wuhan 24

Table S8. Summary of data used to estimate the disease burden and clinical severity of COVID-19 in Wuhan 24

Supplementary file 11: National ILI surveillance network and SARI surveillance in Jingzhou 26
Supplementary file 12: Estimating the number of ILI cases in Wuhan................27

Fig. S4. Number of outpatients at internal medicine departments and paediatric department in Hubei, 2012-2020 30

Fig. S5. Correlation of number of outpatients at internal medicine departments and paediatric department separately with population size and number of hospitals in Hubei, 2012-2017 31

Table S9. Estimated number of outpatients in pediatric and internal medicine departments, and fever clinics, Wuhan, 2012-2020 32

Table S10. Estimated number of ILI cases in Wuhan, from December 1, 2019 through to March 31, 2020 34

Supplementary file 13: Estimating the number of SARI/pneumonia hospitalizations in Wuhan 36

Table S11. Health service index 37

Supplementary file 14: Estimated disease burden of COVID-19 using ILI consultations, and SARI/pneumonia hospitalizations as a reference 39

Table S12. Estimated number ILI consultations, and SARI/pneumonia hospitalizations between December 1, 2019 through March 31, 2020 in Wuhan, China 39

Supplementary file 1. Case definition

COVID-19

As the epidemic evolved, a total of seven versions of case definitions for laboratory-confirmed-cases were issued by the National Health Commission of China, including mild-, moderate-, severe-, and critical-patients. Patients were confirmed by real-time reverse transcription polymerase chain reaction (RT-PCR) or highly homologous to SARS-CoV-2 by viral gene sequencing.[1, 2] Mild cases refer to cases with mild symptoms and no radiographic evidence of pneumonia. Moderate cases refer to cases with fever, respiratory symptoms, and radiographic evidence of pneumonia. Severe cases refer to cases with any breathing problems, finger oxygen saturation, and low PaO2/FiO2 (PaO2 denotes partial pressure of oxygen in arterial blood; FiO2 denotes fraction of inspired oxygen), etc. Critical cases refer to cases having any respiratory failure, shock, and any other organ failure that requires ICU admission.

A clinical diagnosis for COVID-19 cases was only used briefly in Hubei province in the fifth version of case difinition, refer to a suspected case with radiographic evidence of pneumonia.[3] The definition of a suspected case includes having any or no epidemiologic links, and meeting any two of the clinical criteria (fever and/or respiratory symptoms, normal or reduced white blood cell count, or reduced lymphocyte count at early onset of symptoms).

Influenza-like-illness (ILI)

A patient was defined as an ILI case if he/she had a body temperature ≥38 °C with either cough or sore throat, in the absence of an alternative diagnosis.

Severe acute respiratory infection (SARI)

A patient was defined as having SARI if he/she had an elevated temperature (rectal or axillary temperature ≥37.3˚C) and at least one sign or symptom of acute respiratory illness, including cough, sore throat, tachypnea, difficulty breathing, abnormal breath sounds on auscultation, sputum production, hemoptysis, chest pain, or chest radiograph consistent with pneumonia.

Pneumonia

Community-acquired Pneumonia (CAP)[4]

1. Definition

CAP refers to infectious pulmonary parenchymal (including alveolar walls, that is, the interstitial substance of lung in general) inflammation outside the hospital, including pneumonia that develops during the incubation period after a hospital infection with a pathogen with a clear incubation period.

2. Clinical diagnosis of CAP

a. Symptoms onset in the community;

b. Pneumonia-related clinical manifestations;

    1) Newly appeared cough and sputum, or aggravating of pre-existing

respiratory symptoms, with or without purulent phlegm, chest pain, dyspnea and hemoptysis;

2) Fever;
3) Signs of lung consolidation and/or evidence of moist rales;
4) Peripheral blood leucocyte > 10×10^9 /L or < 4×10^9 /L, with or without cell nucleus shift to the left

c. Chest radiology test shows recent patchy infiltrating shadows, consolidation shadows of the lobe or segment, ground glass shadows or interstitial substance change, with or without pleural effusion.

Clinical diagnosis can be established upon compliance with a, c and any one of b, excluding tuberculosis, pulmonary tumor, non-infectious interstitial pulmonary disease, pulmonary edema, pulmonary atelectasis, pulmonary embolism, pulmonary eosinophilic infiltration, and pulmonary vasculitis.

Hospital-acquired Pneumonia (HAP)[5]

1. Definition

HAP refers to the newly appeared pneumonia that occurred 48 hours after the patient was hospitalized without invasive mechanical ventilation and not in the incubation period of pathogen infection.

2. Clinical diagnosis of HAP

a. Pneumonia-related clinical manifestations;

   1) Fever. The temperature is > 38℃;

   2) Purulent airway discharge;

   3) Peripheral blood leucocyte > 10×10^9 /L or < 4×10^9 /L

b. Chest X-ray or CT shows newly appeared or progressive infiltrating shadows, consolidation shadows or ground glass shadows.

Clinical diagnosis can be established upon compliance with b and any one of a.

Supplementary file 2. Description of active screening in Wuhan

To identify the source of infection and treat COVID-19 patients as fast as possible, active community universal screenings of fever cases was performed intensively among the general population in Wuhan on January 24-February 10, and February 17-19, respectively.

Table S1. Active community universal screenings of fever cases among general population in Wuhan

	**1st screening**	**2nd screening**
Duration	Jan 24, 2020 – Feb 10, 2020[6]	Feb 17, 2020 – Feb 19, 2020[7]
Objectives	To check whether the residents had fever and arrange population with fever.	To ensure that all confirmed COVID-19 cases were hospitalized; all suspected cases and patients with fever were tested using RT-PCR; all close contacts of confirmed cases were isolated.
Measures	All individuals were examined for fever. Patients with confirmed or highly suspected COVID-19 were sent to the designated sites for treatment. Suspected cases with fever were kept in the fever clinics for medical observation. Mild cases with fever who could not be identified as suspected patients are sent to designated sites for isolation and medical observation. Cases with fever but negative for SARS-CoV-2 stayed at home for observation.	A 3-day intensive screening was conducted to guarantee the 'Five 100%': 100% of confirmed COVID-19 cases are sent to the designated hospitals; 100% of suspected cases receive RT-PCR test; 100% of patients with fever receive RT-PCR test; 100% of close contacts of confirmed cases are isolated; 100% of the communities and villages adopt 24-hour closed-off management.
Results	Over 4,243,000 families, 10.6 millions residents were screened, with a screening rates of 99.7%. It identified 16,568 confirmed cases, 14,596 suspected cases, 17,473 close contacts, and 7,961 general fever patients.[8]	As of Feb 19, 2020, most districts in Wuhan have screened all the residents. In Hongshan District, Caidian District, Huangpi District and East Lake Senic Area of Wuhan, 30, 138, 0 and 45 lab-confirmed cases were identified respectively. To sum up, a total of 213 confirmed cases were identified.[7]
Source of information	Official website of Health Commission of Hubei province and Wuhan, as well as that of Hubei province government, etc.	

Supplementary file 3: Disease burden and clinical severity of COVID-19 in Wuhan

Table S2. Summary of disease burden and clinical severity of COVID-19 in baseline and sensitivity analyses

Age (years) ξ	Reported cases	Reported number of deaths	Estimated disease burden (per 100,000 persons) (n, 95%CI)				Estimated clinical severity (%, 95%CI)				
			Rates of symptomatic cases	Rates of medical consultation	Hospitalization rates	Mortality rates	sCFR	mCFR	HFR	sCHR	mCHR
**Baseline**											
0-19	828	4	55 (50-60)	43 (43-44)	32 (31-32)	0.2 (0.1-0.5)	0.36 (0.33-0.40)	0.46 (0.45-0.47)	0.62 (0.62-0.63)	58.18 (54.17-63.00)	73.56 (73.26-73.86)
20-39	9,207	95	448 (404-515)	257 (250-268)	198 (193-206)	2.4 (2.0-3.0)	0.55 (0.47-0.61)	0.95 (0.91-0.98)	1.23 (1.19-1.27)	44.25 (40.00-47.77)	77.00 (76.92-77.32)
40-59	18,953	635	957 (859-1,106)	598 (577-621)	455 (440-473)	18.9 (17.5-20.4)	1.97 (1.71-2.20)	3.16 (3.04-3.27)	4.15 (3.99-4.29)	47.52 (42.74-51.21)	76.12 (76.08-76.29)
≥60	21,345	3,135	2,386 (2,047-3,188)	1,473 (1,418-1,542)	1,099 (1,065-1,148)	216.8 (209.3-224.5)	9.09 (6.80-10.59)	14.72 (14.06-15.29)	19.72 (18.89-20.36)	46.09 (36.01-52.03)	74.65 (74.44-75.12)
All	50,333	3,869	796 (703-977)	489 (472-509)	370 (358-384)	36.2 (35.0-37.3)	4.54 (3.70-5.14)	7.40 (7.10-7.66)	9.77 (9.41-10.10)	46.48 (39.33-50.93)	75.72 (75.41-75.84)

**Scenario i)**†										
0-19	828	4	55 (55-65)	40 (40-41)	27 (26-27)	0.2 (0.1-0.5)	0.36 (0.31-0.36)	0.49 (0.49-0.50)	0.74 (0.74-0.75)	48.18 (41.54-49.09)	66.25 (65.85-66.67)
20-39	9,207	95	538 (471-641)	234 (229-245)	170 (165-175)	2.4 (2.0-3.0)	0.45 (0.38-0.52)	1.04 (1.00-1.07)	1.44 (1.40-1.48)	31.58 (27.31-34.97)	71.91 (71.58-72.53)
40-59	18,953	635	1,118 (969-1,353)	541 (523-565)	392 (378-407)	18.9 (17.5-20.4)	1.69 (1.40-1.95)	3.49 (3.34-3.61)	4.81 (4.64-5.00)	35.11 (30.11-38.96)	72.16 (72.11-72.53)
≥60	21,345	3,135	2,794 (2,261-4,052)	1,328 (1,279-1,383)	947 (913-989)	216.8 (209.3-224.5)	7.76 (5.35-9.59)	16.33 (15.68-16.95)	22.88 (21.92-23.75)	33.91 (24.40-40.37)	71.35 (71.35-71.50)
All	50,333	3,869	935 (792-1,218)	443 (427-461)	318 (308-330)	36.2 (35.0-37.3)	3.87 (2.97-4.57)	8.16 (7.85-8.47)	11.38 (10.96-11.76)	34.00 (27.09-38.84)	71.73 (71.60-71.99)
**Scenario ii)**†											
0-19	828	4	35 (35-40)	26 (25-27)	14 (14-15)	0.2 (0.1-0.5)	0.51 (0.45-0.51)	0.69 (0.66-0.70)	1.23 (1.19-1.27)	40.00 (37.50-41.43)	55.56 (54.90-55.77)
20-39	9,207	89	353 (309-420)	162 (154-172)	103 (98-111)	2.3 (1.9-2.8)	0.65 (0.55-0.74)	1.42 (1.33-1.49)	2.23 (2.07-2.35)	29.20 (26.38-31.67)	63.49 (63.33-64.18)
40-59	18,953	599	749 (654-901)	392 (372-416)	250 (235-268)	17.8 (16.4-19.3)	2.38 (1.98-2.72)	4.54 (4.28-4.79)	7.13 (6.66-7.59)	33.33 (29.70-35.91)	63.64 (63.20-64.29)

≥60	21,345	2,957	1,943 (1,604-2,745)	1,030 (975-1,099)	657 (622-705)	204.5 (197.2-212.0)	10.52 (7.45-12.75)	19.85 (18.60-20.97)	31.13 (28.99-32.86)	33.81 (25.69-38.79)	63.83 (63.76-64.15)
All	50,333	3,649	634 (540-814)	327 (309-347)	208 (196-222)	34.1 (33.0-35.2)	5.38 (4.19-6.31)	10.43 (9.84-11.02)	16.44 (15.40-17.38)	32.74 (27.21-36.33)	63.44 (63.43-63.88)
**Scenario iii)**[†]											
0-19	828	4	50 (50-55)	44 (43-45)	32 (31-32)	0.2 (0.1-0.5)	0.40 (0.36-0.40)	0.45 (0.44-0.46)	0.62 (0.62-0.63)	63.00 (59.09-64.00)	72.41 (72.22-72.73)
20-39	9,207	95	414 (381-463)	263 (255-273)	198 (193-206)	2.3 (1.9-2.8)	0.59 (0.53-0.64)	0.93 (0.90-0.96)	1.23 (1.19-1.27)	47.83 (44.44-50.68)	75.49 (75.47-75.76)
40-59	18,953	635	883 (809-993)	609 (589-636)	455 (440-473)	17.8 (16.4-19.3)	2.14 (1.90-2.33)	3.10 (2.97-3.21)	4.15 (3.99-4.29)	51.52 (47.60-54.41)	74.63 (74.30-74.75)
≥60	21,345	3,135	2,074 (1,874-2,448)	1,507 (1,445-1,577)	1,099 (1,065-1,148)	204.5 (197.2-212.0)	10.45 (8.86-11.57)	14.38 (13.75-15.00)	19.72 (18.89-20.36)	53.00 (46.89-56.83)	72.94 (72.81-73.68)
All	50,333	3,869	719 (654-821)	499 (481-521)	370 (358-384)	34.1 (33.0-35.2)	5.03 (4.41-5.53)	7.25 (6.95-7.51)	9.77 (9.41-10.10)	51.50 (46.81-54.71)	74.16 (73.79-74.37)
**Scenario iv)**[†]											
0-19	828	4	55 (55-65)	43 (42-43)	32 (31-32)	0.2 (0.1-0.5)	0.36 (0.31-0.36)	0.47 (0.46-0.47)	0.62 (0.62-0.63)	57.27 (50.00-58.18)	74.42 (74.12-74.71)

20-39	9,207	95	499 (438-600)	252 (245-263)	198 (193-206)	2.3 (1.9-2.8)	0.49 (0.41-0.56)	0.97 (0.93-1.00)	1.23 (1.19-1.27)	39.69 (34.33-44.12)	78.57 (78.43-78.95)
40-59	18,953	635	1,079 (937-1,332)	586 (565-609)	455 (440-473)	17.8 (16.4-19.3)	1.75 (1.42-2.02)	3.22 (3.10-3.34)	4.15 (3.99-4.29)	42.15 (35.49-46.98)	77.66 (77.56-77.89)
≥60	21,345	3,135	3,174 (2,406-5,698)	1,445 (1,390-1,507)	1,099 (1,065-1,148)	204.5 (197.2-212.0)	6.83 (3.80-9.01)	15.00 (14.38-15.60)	19.72 (18.89-20.36)	34.64 (20.15-44.25)	76.15 (76.08-76.62)
All	50,333	3,869	960 (789-1,419)	479 (463-498)	370 (358-384)	34.1 (33.0-35.2)	3.77 (2.55-4.58)	7.56 (7.26-7.82)	9.77 (9.41-10.10)	38.56 (27.08-45.38)	77.34 (77.11-77.37)

[ξ] Age profile of officially reported cases was obtained in a Wuhan study which includes 32,583 laboratory-confirmed COVID-19 cases as of March 8, 2020.[9] While age profile of fatal cases was obtained from China CDC Weekly report.[10]

[†] Scenario i): we assumed moderate cases had the same health seeking behavior as mild cases, i.e., only a proportion of moderate and mild cases sought medical assistance; scenario ii): clinically-diagnosed cases were excluded; iii): using the upper limit of 95%CI of the probability of seeking medical care; iv): using the lower limit of 95%CI of the probability of seeking medical care

Supplementary file 4: Disease burden of COVID-19, influenza pandemic in 2009 and seasonal influenza

Table S3. Summary of disease burden of COVID-19, influenza pandemic in 2009 and seasonal influenza

Parameters	Authors (year)	Locations	Influenza season	Methods	Influenza type	Results[ξ]
Influenza-associated excess ILI outpatient consultations	Feng L (2020)[11]	China	2009-10	Generalized additive regression models were fitted to estimate influenza-associated excess ILI outpatient burden.	A(H1N1)pdm	per 1,000 person-years (mean, 95%CI)[†]<br>0-14 yrs: 10.8 (5.7-16.2); 15-59 yrs: 6.9 (5.4-8.3); ≥60 yrs: 1.3 (0.1-2.8); overall: 6.8 (5.5-8.1)
			2006-15		Seasonal influenza	per 1,000 person-years (mean, 95%CI)<br>0-14 yrs: 4.5 (1.3-7.9); 15-59 yrs: 2.3 (1.4-3.3); ≥60 yrs: 1.1 (0.3-2.0); overall: 2.5 (1.5-3.6)
Medically-attended rate	Fowlkes A (2015)[12]	USA	Oct 2009-Jul 2010	Influenza Incidence Surveillance Project using population-based surveillance: the incidence of visits for influenza-like illness was calculated using the size of the patient population, and the incidence attributable to influenza was extrapolated from the proportion of patients with positive tests each week.	A(H1N1)pdm	per 1,000 person-seasons (mean, 95%CI)<br><2 yrs: 13.9 (9.3-18.8); 2-4 yrs: 20.8 (17.4-24.5);<br>5-17 yrs: 27.1 (25.0-29.2); 18-24 yrs: 13.4 (11.5-15.2);<br>25-49 yrs: 5.5 (4.7-6.4); 50-64 yrs: 2.9 (2.1-3.7);<br>≥65 yrs: 0.9 (0.6-1.3); overall: 10.3 (9.6-10.8)
			2012-13		Seasonal influenza	per 1,000 person-seasons (mean, 95%CI)<br><2 yrs: 13.2 (11.2-15.2); 2-4 yrs: 27.4 (24.4-30.4);<br>5-17 yrs: 25.5 (24.0-26.9); 18-24 yrs: 5.9 (5.4-6.4);<br>25-49 yrs: 6.5 (6.0-7.0); 50-64 yrs: 4.9 (4.3-5.7);<br>≥65 yrs: 3.5 (2.9-4.2); overall: 10.7 (10.3-11.1)
Hospitalization rate	Shrestha SS (2011)[13]	USA	Apr 2009-Apr 2010	The weekly population reports of laboratory-confirmed pH1N1-related hospitalizations were used to calculate a range of hospitalizations per 100,000 population in a model, then extrapolate those hospitalization rates to the 50 states in US.	A(H1N1)pdm	per 100,000 person-seasons (median, range)<br>0-17 yrs: 117.4 (83.5-172.4); 18-64 yrs: 83.8 (59.6-123.0);<br>≥65 yrs: 70.1 (49.9-103.0); overall: 90.2 (64.2-132.4)
Hospitalization rate	Yu H (2014)[14]	Jingzhou, China	2011-12	The rate of influenza-associated severe acute respiratory infection (SARI) hospitalization was estimated based on population-based surveillance.	Seasonal influenza	per 100,000 person-years (median, range)<br>0-14 yrs: 1,116.5 (1,083.1-1159.2); 15-64 yrs: 16.9 (15.7-17.4);<br>≥65 yrs: 88.7 (85.3-90.5); overall: 142.2 (140.2-144.2)
Hospitalization	Palekar RS	USA	2010-13	The influenza-associated hospitalizations rate was estimated	Seasonal	per 100,000 persons (mean, 95%CI)

rate	(2019)[15]			by multiplying the monthly number of respiratory hospitalizations by the monthly proportion of influenza-positive samples and dividing by the census population.	influenza	0-4 yrs: 86 (39-190); 5-64 yrs: 83 (46-150); ≥65 yrs: 487 (245-971)
Hospitalization rate	Reed C (2015)[16]	USA	2012-13	Population-based rates of influenza-associated hospitalizations from 2010–2013 were extrapolated to the U.S. population from FluSurv-NET and corrected for under-detection.	Seasonal influenza	per 100,000 persons (mean, 95%CI) overall: 202 (143-260)
Mortality rate	Dawood FS (2012)[17]	USA	Apr 2009-Apr 2010	The crude respiratory mortality rates associated with the 2009 pandemic influenza A H1N1 strain were calculated using the cumulative (12 months) virus-associated symptomatic attack rates (sAR) from 12 countries and symptomatic case fatality ratios (sCFR) from five high-income countries. Second, the estimated base mortality in each risk group was adjusted using a risk group-specific respiratory mortality multiplier. Then the number of respiratory deaths associated with 2009 pandemic influenza A H1N1 for each age and country-risk group was estimated by multiplying sAR, sCFR, RMM, and age group population.	A(H1N1)pdm	per 100,000 persons (25% and 75% percentiles) 0-17 yrs: 0.5-1.6; 18-64 yrs: 1.0-3.3; ≥65 yrs: 1.8-9.0; overall: 1.0-3.6
Excess mortality rate	Li L (2019)[18]	China	2010-15	The influenza-associated excess respiratory deaths were estimated by subtracting the predicted number of respiratory deaths assuming that the influenza virus activity proxy was 0 from the predicted number of respiratory deaths using the observed influenza virus activity proxy.	Seasonal influenza	per 100,000 person-seasons (mean, 95%CI) <60 yrs: 1.5 (1.1-1.9); ≥60 yrs: 38.5 (36.8-40.2); overall: 6.5 (6.3-6.8)
Excess mortality rate	Iuliano AD (2018)[19]	USA	1999-2015	The country-specific influenza-associated respiratory excess mortality rates (EMR) were estimated for 33 countries using time series log-linear regression models with vital death records and influenza surveillance data.	Seasonal influenza	per 100,000 persons (median, 95%CrI) <65 yrs: 0.6 (0.4-0.7); 65-74 yrs: 8.6 (6.6-10.6); ≥75 yrs: 49.3 (37.1-61.9); overall: 4.1 (1.9-12.7)

[ξ]CI, confidence interval; CrI, credibility interval. [†]The study presented the ILI conculstations by age group associated with all subtypes/ lineages of influenza in 2009-2010, and the overall estimates associated with each subtype/ lineages. Considering that A(H1N1)pdm dominated in 2009-2010, the results of A(H1N1)pdm associated with ILI consultations by age group were calculated with an assumption that the proportion of A(H1N1)pdm associated consultations in all influenza associated consultations was the same in each age group.

Supplementary file 5: Summary of severity of the COVID-19 pandemic, and 1918 and 2009 influenza pandemics

Table S4. Severity of the 1918 and 2009 influenza pandemics

Parameter	1918 influenza pandemic (mean)	2009 influenza pandemic (mean/median, 95%CI)
Symptomatic case fatality risk (sCFR) (risk of death among symptomatic infections)	**USA**[#20]<br>Overall: 1.61%;<br><1 yrs: 5.17%; 1-4 yrs: 1.76%<br>5-9 yrs: 0.45%; 10-14 yrs: 0.51%;<br>15-19 yrs: 0.91%; 20-24 yrs: 1.83%;<br>25-29 yrs: 2.96%; 30-34 yrs: 2.30%;<br>35-39 yrs: 2.19%; 40-44 yrs: 1.54%;<br>45-49 yrs: 1.35%; 50-54 yrs: 1.51%;<br>55-59 yrs: 0.90%; 60-64 yrs: 2.82%;<br>65-69 yrs: 4.33%; 70-74 yrs: 5.25%;<br>≥75 yrs: 3.03%<br>Age-standardized risk[††]: 1.95%<br>**USA**[##21]<br>Overall: 1.98%	**USA**[†22]<br>Overall: 0.048% (0.026, 0.096);<br>0-4 yrs: 0.026% (0.006, 0.092);<br>5-17 yrs: 0.010% (0.003, 0.031);<br>18-64 yrs: 0.159% (0.066, 0.333);<br>≥65 yrs: 0.090% (0.008, 1.471)<br>Age-standardized risk[††]: 0.121% (0.047, 0.397)
Hospitalization fatality risk (HFR) (risk of death among infections that require hospitalization for medical reasons, not only for case isolation)	--	**North America** [23]<br>Overall: 2.6% (1.6, 3.9);<br>≤19 yrs: 0.8% (0.5, 1.1);<br>20-64 yrs: 5.4% (3.5, 7.5);<br>≥65 yrs: 10.7% (5.3, 17.6)<br>Age-standardized risk[††]: 5.0% (3.1, 7.3)
Symptomatic cases hospitalization risk (sCHR) (risk of hospitalization among symptomatic infections)	--	**USA**[†22]<br>Overall: 1.44% (0.83, 2.64);<br>0–4 yrs: 2.45% (1.10, 5.56);<br>5–17 yrs: 0.61% (0.27, 1.34);<br>18–64 yrs: 3.00% (1.35, 5.92);<br>≥65 yrs: 1.84% (0.21, 25.38)<br>Age-standardized risk[††]: 2.5% (1.0, 7.3)

[#] The surveys were made mostly during December, after the first wave of the epidemic had subsided, but before the second wave started.

[##] The data were collected from November 20, 1918 to December 15, 1918, covering only the fall wave from September 1, 1918 to November 30, 1918.

[†] Based on a combination of data from New York City and Milwaukee, and survey data on the frequency of medical attendance of symptomatic cases.

[††]Age-standardized risk were estimated by using age profile of Wuhan population as a reference.

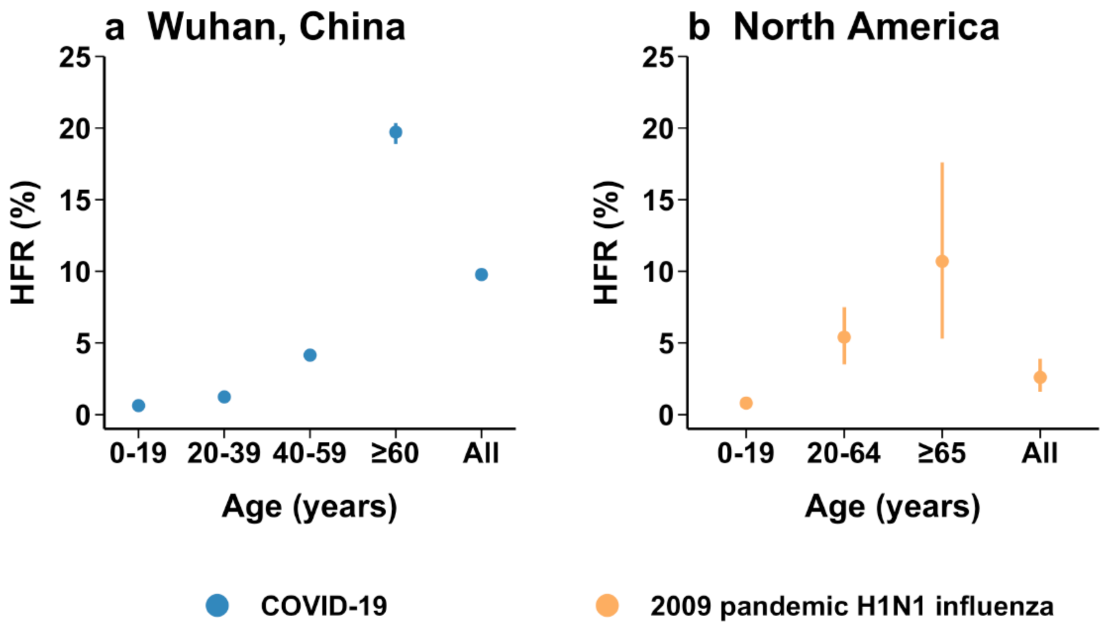

Fig. S1. Hospitalization-fatality risk (HFR)

a: HFR associated with COVID-19 in Wuhan, China (mean, 95%CI). The error bars presented 95% CI as estimated using Monte Carlo sampling (10,000 samples from Binomial distributions).

b: HFR associated with 2009 pandemic H1N1 influenza, North America (mean,95%CI) [23]

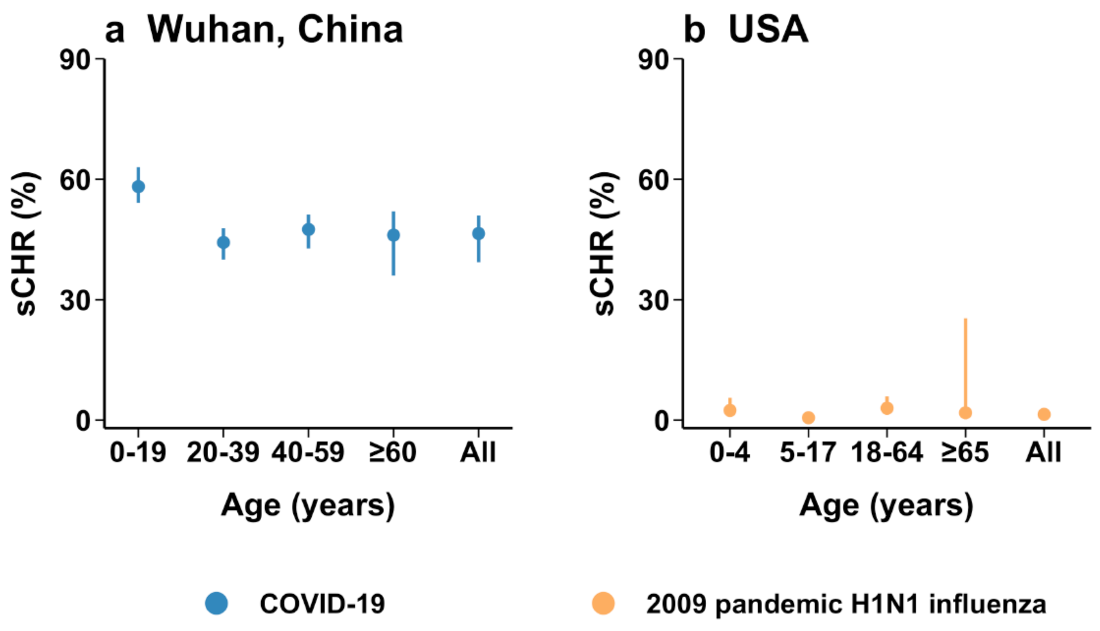

Fig. S2. Symptomatic case-hospitalization risk (sCHR)

a: sCHR associated with COVID-19 in Wuhan, China (mean, 95%CI). The error bars presented 95% CI as estimated using Monte Carlo sampling (10,000 samples from Binomial distributions).

b: sCHR associated with 2009 pandemic H1N1 influenza, USA (median,95%CI) [22]

Supplementary file 6: Estimated disease burden of COVID-19 based on ILI consultations, and SARI/pneumonia hospitalizations

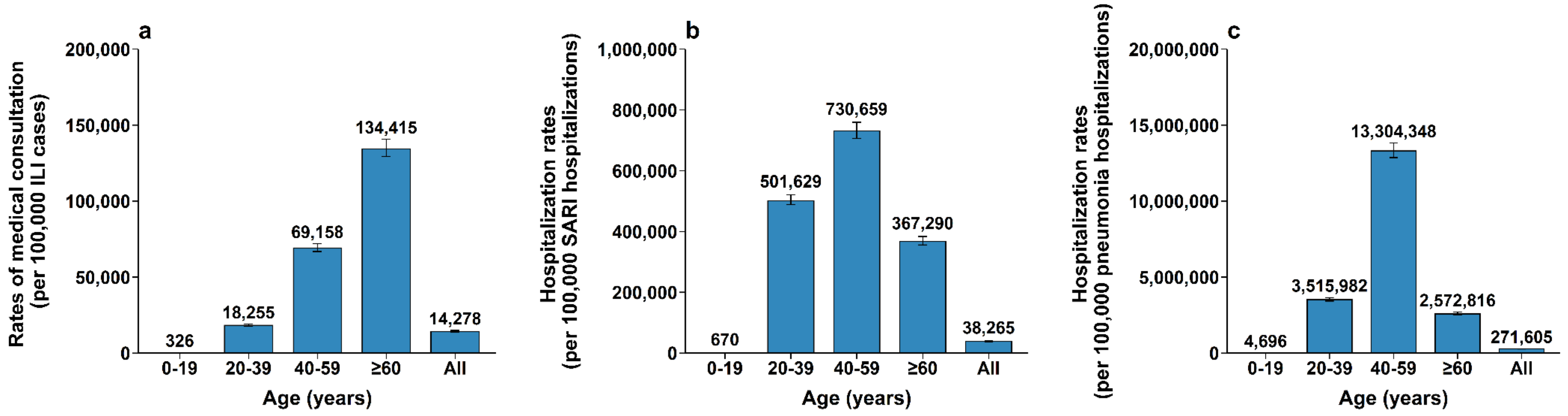

Fig. S3. Estimated disease burden of COVID-19 based on ILI consultations, and SARI/pneumonia hospitalizations between December 1, 2019 through March 31, 2020 in Wuhan, China (mean, 95%CI)

A: rates of medically attended influenza-like-illness (ILI) associated with SARS-CoV-2 infections; B: rates of hospitalized severe acute respiratory infection (SARI) associated with SARS-CoV-2 infections; C: rates of hospitalized pneumonia associated with SARS-CoV-2 infections. The error bars presented 95% CI as estimated using Monte Carlo sampling (10,000 samples from Binomial distributions).

Supplementary file 7: Summary of disease burden estimates of COVID-19 in Canada and the US

Table S5. Disease burden estimates of COVID-19 in Canada and the US

References	Country	Study periods	Testing strategy for COVID-19 cases	Outcomes	Locations	Results
Bignami S. et al., (medRxiv)[24]	Canada	As of May 31	• Testing strategy varied across provinces (e.g. Québec and Ontario preferred to test symptomatic cases until the end of March, while other provinces preferred to test healthcare staff);<br>• Did not account for the sensitivity of RT-PCR and healthcare seeking behaviors	Rate of symptomatic cases (per 100,000 persons)	Nation	0-19 yrs: 49; 20-39 yrs: 219; 40-59 yrs: 265; 60-79 yrs: 210; ≥80 yrs: 1,024; overall: 228
					Québec	0-19 yrs: 104; 20-39 yrs: 512; 40-59 yrs: 604; 60-79 yrs: 440; ≥80 yrs: 2,592; overall: 534
					Ontario	0-19 yrs: 36; 20-39 yrs: 192; 40-59 yrs: 232; 60-79 yrs: 200; ≥80 yrs: 814; overall: 200
				Hospitalization rate (per 100,000 persons)	Nation	0-19 yrs: 1; 20-39 yrs: 6; 40-59 yrs: 18; 60-79 yrs: 44; ≥80 yrs: 180; overall: 23
					Québec	0-19 yrs: 3; 20-39 yrs: 13; 40-59 yrs: 33; 60-79 yrs: 83; ≥80 yrs: 409; overall: 50
					Ontario	0-19 yrs: 1; 20-39 yrs: 6; 40-59 yrs: 21; 60-79 yrs: 49; ≥80 yrs: 168; overall: 25
				Mortality rate (per 100,000 persons)	Nation	0-19 yrs: 0; 20-39 yrs: 0; 40-59 yrs: 2; 60-79 yrs: 24; ≥80 yrs: 313; overall: 19
					Québec	0-19 yrs: 0; 20-39 yrs: 0; 40-59 yrs: 3; 60-79 yrs: 58; ≥80 yrs: 809; overall: 52
					Ontario	0-19 yrs: 0; 20-39 yrs: 0; 40-59 yrs: 2; 60-79 yrs: 22; ≥80 yrs: 251; overall: 16

Stokes E.K. et al., (MMWR)[25]	USA	Jan 22 to May 30	• Not every case in the community was captured through testing and asymptomatic cases were not captured well in case surveillance.<br>• According to CDC, only the following populations, including individuals with signs or symptoms consistent with COVID-19, asymptomatic individuals with suspected exposure to SARS-CoV-2 and other individuals being tested for public health surveillance, were preferentially tested.[26]<br>• Did not account for sensitivity of RT-PCR or healthcare seeking behaviors	Rate of symptomatic cases (per 100,000 persons)	Nation	0-9 yrs: 51.1; 10-19 yrs: 117.3;<br>20-29 yrs: 401.6; 30-39 yrs: 491.6;<br>40-49 yrs: 541.6; 50-59 yrs: 550.5;<br>60-69 yrs: 478.4; 70-79 yrs: 464.2;<br>≥80 yrs: 902.0; overall: 403.6
				Hospitalization rate (per 100,000 persons)	Nation	**No. hospitalizations:**<br>0-9 yrs: 848; 10-19 yrs: 1,234;<br>20-29 yrs: 6,704; 30-39 yrs:12,570;<br>40-49 yrs: 19,318; 50-59 yrs:31,588;<br>60-69 yrs: 39,422; 70-79 yrs: 38,544;<br>≥80 yrs: 37,145; overall: 184,673<br><br>**Population size[27]:**<br>0-9 yrs:40,215,846; 10-19 yrs: 42,858,117<br>20-29 yrs: 46,557,394; 30-39 yrs: 44,378,948<br>40-49 yrs: 40,745,901; 50-59 yrs: 43,021,065<br>60-69 yrs: 38,237,107; 70-79 yrs: 23,293,643<br>≥80 yrs: 13,074,699; overall: 332,382,720<br><br>**Hospitalization rate*:**<br>0-9 yrs: 2.1; 10-19 yrs: 2.9;<br>20-29 yrs:14.4; 30-39 yrs: 28.3;<br>40-49 yrs: 47.4; 50-59 yrs:73.4;<br>60-69 yrs: 103.1; 70-79 yrs: 153.9;<br>≥80 yrs: 284.1; overall: 55.6
Lewnard, J.A. et al., (BMJ)[28]	USA	As of April 22, 2020	• In early April, the daily testing number in California was low (around 2,000 a day).[29] COVID-19 testing in California has rapidly expanded over the past three months. The California Department of Public Health recommends first prioritizing testing of hospitalized individuals with signs or symptoms of COVID-19	Hospitalization rate (per 100,000 persons)	Northern California	0-9 yrs: 0; 10-19 yrs: 1.1;<br>20-29 yrs: 5.3; 30-39 yrs: 6.8;<br>40-49 yrs: 17.4; 50-59 yrs: 25.2;<br>60-69 yrs: 29.2; 70-79 yrs: 39.1;<br>≥80 yrs: 74.3
					Southern	0-9 yrs: 0; 10-19 yrs: 0.7;

			infection followed by testing of other symptomatic individuals and higher risk asymptomatic individuals and then other asymptomatic individuals when certain conditions exist.[30]		California	20-29 yrs: 6.4; 30-39 yrs: 16.0;<br>40-49 yrs: 25.7; 50-59 yrs: 38.0;<br>60-69 yrs: 49.3; 70-79 yrs: 56.8;<br>≥80 yrs: 90.4
			• Testing priority populations for the Period of May-July 2020 in Washington (highest to lowest): a. Persons with COVID-19-like symptoms; b. Close contacts of persons with COVID-19-like symptoms; c. Those in congregate settings with one or more active cases.[31]		Washington state	0-9 yrs: 0; 10-19 yrs: 0;<br>20-29 yrs: 2.1; 30-39 yrs: 7.9;<br>40-49 yrs: 13.5; 50-59 yrs: 18.7;<br>60-69 yrs: 27.6; 70-79 yrs: 45.3;<br>≥80 yrs: 46.6
Garg S. et al., (MMWR)[32]	USA	As of Jul 11, 2020	• The testing strategy varied slightly by state, but a large number of cases were not tested before May 2020. Since then, some states rapidly expanded COVID-19 testing, such as California, New York, etc. New York State has aggressively expanded COVID-19 diagnostic testing capacity after July 1st.[30, 33]	Hospitalization rate (per 100,000 persons)	14 States, USA	0-4 yrs: 10.6; 5-17 yrs: 5.3; 18-29 yrs:42.7; 30-39 yrs: 70.9; 40-49 yrs: 112.5; 50-64 yrs: 171.8; 65-74 yrs: 236.1; 75-84 yrs: 382.1; ≥85 yrs: 607.3; overall 113.6
New York City Department of Health and Mental Hygiene COVID-19 Response Team, (MMWR)[34]	USA	Mar 11 to May 2	• Although New York State has aggressively expanded COVID-19 diagnostic testing capacity after July 1st, a large number of cases were not tested before, especially in the worst-hit area in USA.[33]	Excess deaths	New York	**Estimated excess all-cause deaths:**<br>24,172 (95% CI: 22,980–25,364);<br>Laboratory-confirmed COVID-19–associated deaths: 13,831;<br>Probable COVID-19–associated deaths: 5,048;<br>Deaths might have been directly or indirectly attributable to the pandemic: 5,293
Weinberger D.M. et al., (JAMA Intern Med)[35]	USA	Mar 1 to May 30	• Not every case in the community was captured through testing and asymptomatic cases were not captured well in case surveillance.<br>• According to CDC, only the following populations, including individuals with signs or symptoms consistent with COVID-19, asymptomatic individuals with suspected exposure to SARS-CoV-2 and other individuals being tested for public health surveillance, were preferentially tested.[26]	Excess deaths	Nation	**Estimated excess all-cause deaths:**<br>122,300 (Poisson 95% prediction interval 116,800-127,000)<br><br>**Estimated excess pneumonia/influenza/ COVID-19 deaths:**<br>94,130 (Poisson 95% prediction interval: 92,350 to 95,690)

*we calculated the hospitalization rate using the number of hospitalizations and corresponding population size.

Supplementary file 8: Reported and age-standardized rates of 2009 pandemic influenza and seasonal influenza in USA and China

Table S6. Reported and age-standardized rates of 2009 pandemic influenza and seasonal influenza in USA and China

	USA (n, 95%CI)		China (n, 95%CI)	
	Reported†	Age-standardized ††	Reported†	Age-standardized ††
Medical consultation rates				
Pandemic H1N1 influenza†††[11, 12]	1030 (960, 1080)	840.8 (720.1, 966.5)	680 (550, 810)	654.9 (472.9, 854.4)
Seasonal influenza[11, 12]	1070 (1030, 1110)	829.9 (756.7, 907.0)	250 (150, 360)	242.2 (120.7, 375.1)
Hospitalization rates				
Pandemic H1N1 influenza[13]	90.2 (64.2, 132.4)	89.3 (63.5, 131.1)	-	-
Seasonal influenza[16]	202 (143, 260)	126.9 (67.2, 241.1)	-	-
Mortality rates				
Pandemic H1N1 influenza[17]†††	(1.0, 3.6)	(1.0, 3.7)	-	-
Seasonal influenza[18, 19]	4.1 (1.9, 12.7)	3.2 (2.3, 3.9)	6.5 (6.3, 6.8)	7.5 (6.9, 8.1)

† Reported rates were extracted from published literatures.

†† Standardized using age profile of Wuhan population as a reference.

†††Only 95%CI for mortality rates were reported in USA, and no mean was reported.

Supplementary file 9: Adjustment for RT-PCR sensitivity

The officially reported laboratory-confirmed COVID-cases were mainly tested using RT-PCR. A study in Wuhan found the RT-PCR sensitivity decreased with the number of days since symptom onset.[36] (Table S2).

Table S7. Time interval from symptom onset to diagnosis in Wuhan

	Before Feb 1	Feb 2 to 16	Feb 17 to Mar 31	Total
Interval from symptom onset to diagnosis (days)[ξ] (median, IQR)	12 (8-17)	6 (3-10)	3 (1-6)	9 (5-14)
PCR sensitivity[§] (mean, 95%CI)	0.688 (0.624, 0.745)	0.979 (0.939, 0.993)	0.979 (0.939, 0.993)	0.688 (0.624, 0.745)

[ξ] Intervals from symptom onset to diagnosis were obtained in a Wuhan study which includes 32,583 laboratory-confirmed COVID-19 cases as of March 8, 2020.[9]

[§] A study in Wuhan reported the highest sensitivity of RT-PCR test within 7 days (97.9%) post symptom onset, followed by 68.8%, 36.3%, 30.0% and 26.3% at day 8-14, day 15-21, day 22-28, and >28 days respectively.[36]

Supplementary file 10: Summary of data used to estimate the disease burden and clinical severity of COVID-19 in Wuhan

Table S8. Summary of data used to estimate the disease burden and clinical severity of COVID-19 in Wuhan

Parameters	Variables	Before Feb 1	Feb 2- Feb 16	Feb 17-Mar 8	Mar 9-Apr 24	Overall	Data source
COVID-19 cases	Number of confirmed COVID-19 cases by period, until March 8, 2020 (based on symptom onset date)	19,521	9,972	3,090	-	-	Published article, in which the data were extracted from the National Notifiable Disease Report System[37]; and individual level data available on GitHub at https://github.com/chaolongwang/SAPHIRE
	Number of confirmed COVID-19 cases after March 8 (based on report date)	-	-	-	60	-	Wuhan Municipal Health Commission. Data were extracted from the municipal Notifiable Disease Report System[38]
	Revised number of confirmed COVID-19 cases after systematic verification between late March and middle April	-	-	-	-	325	Government affiliated medias[39, 40]
	Number of clinically-diagnosed COVID-19 cases by period (based on symptom onset date)	9,993	7,091	281	-	-	We obtained the aggregated data from the corresponding author of published article[9]
	Number of COVID-19 deaths	-	-	-	-	3,869	Wuhan Municipal Health Commission. Data were extracted from the municipal Notifiable Disease Report System
	Number of confirmed cases captured by two active community screenings	-	-	-	-	16,781	Government affiliated medias[7, 8]
Age profiles	Age distribution of confirmed COVID-19 cases (%)	0–19 yrs: 0.5<br>20–39 yrs: 17.2<br>40–59 yrs: 38.2<br>≥60 yrs: 44.1	0–19 yrs: 2.1<br>20–39 yrs: 18.9<br>40–59 yrs: 37.7<br>≥60 yrs: 41.3	0–19 yrs: 7.2<br>20–39 yrs: 23.5<br>40–59 yrs: 33.9<br>≥60 yrs: 35.4	-	-	Published article, in which the data were extracted from the National Notifiable Disease Report System[9]

	Age distribution of clinically-diagnosed COVID-19 cases (%)	0–19 yrs: 0.8<br>20–39 yrs: 20.4<br>40–59 yrs: 41.6<br>≥60 yrs: 37.2	0–19 yrs: 3.3<br>20–39 yrs: 22.6<br>40–59 yrs: 37.6<br>≥60 yrs: 36.5	0–19 yrs: 10.7<br>20–39 yrs: 20.6<br>40–59 yrs: 34.2<br>≥60 yrs: 34.5	-	-	We obtained the aggregated data from the corresponding author of published article[9]
	Age distribution of COVID-19 deaths (%)	-	-	-	-	0–19 yrs: 0.1<br>20–39 yrs: 2.4<br>40–59 yrs: 16.4<br>≥60 yrs: 81.0	Published article, in which the data were extracted from the National Notifiable Disease Report System[10]
Clinical severity	Clinical severity of confirmed and clinically-diagnosed COVID-19 cases (%)	Mild: 45.4<br>Moderate: 27.2<br>Severe and critical: 27.4	Mild: 49.8<br>Moderate: 34.3<br>Severe and critical: 15.9	Mild: 57.6<br>Moderate: 32.1<br>Severe and critical: 10.3	-	-	Published article, in which the data were extracted from the National Notifiable Disease Report System[9]
	Clinical severity of clinically-diagnosed COVID-19 cases (%)	Moderate: 50.0<br>Severe and critical: 50.0	Moderate: 68.3<br>Severe and critical: 31.7	Moderate: 75.7<br>Severe and critical: 24.3	-	-	We obtained the aggregated data from the corresponding author of published article[9]
Healthcare seeking behaviors	The proportion of persons who sought medical care for acute respiratory infections during the COVID-19 pandemic (%) (mean, 95%CI)	-	-	-	-	3–17 yrs: 66.1 (53.8-78.6)<br>18–39 yrs: 28.0 (21.1-34.8)<br>40–59 yrs: 34.0 (24.7-43.3)<br>≥60 yrs: 35.1 (18.9-51.4)	Population-based telephone and online survey[41]
Test performance	Sensitivity of RT-PCR assays	Supplementary file 9					Published article[36]
Time interval	Interval between symptom onset and laboratory testing	Supplementary file 9					Published article, in which the data were extracted from the National Notifiable Disease Report System [9]

$^{\xi}$ Clinical severity of clinically-diagnosed COVID-19 cases were assumed to be consistent with confirmed cases.

Supplementary file 11: National ILI surveillance network and SARI surveillance in Jingzhou

National ILI surveillance network

The National ILI surveillance network was established to monitor the activity of, as well as antigenic and genetic changes in, seasonal influenza viruses in mainland China. This network includes 408 provincial- and prefecture-level CDCs and 554 sentinel hospitals situated in 31 provinces. All sentinel hospitals report the weekly number of ILI cases who seek medical care in their sentinel outpatient departments (including internal medicine department, pediatric department, and fever department). In each sentinel hospital, respiratory specimens are collected daily from the first one or two reported ILI cases and sent to local CDCs for influenza virus testing using virus isolation and/or reverse transcription polymerase chain reaction (RT-PCR).[11, 18]

SARI surveillance in Jingzhou, Hubei province

To monitor severe disease caused by influenza, SARI Surveillance was conducted at four hospitals in two districts of Jingzhou City, Hubei province in 2010-2012. Surveillance included the majority of hospitalizations for all discharge diagnoses or for influenza-associated primary discharge diagnoses. Nasopharyngeal swabs from SARI patients were collected within 24 hours of admission and tested for influenza virus using real-time reverse transcription PCR (rRT-PCR).[14]

Supplementary file 12: Estimating the number of ILI cases in Wuhan

We estimated the number of ILI cases in Wuhan from Dec.1, 2019 to Mar. 31, 2020, using the formula below.

$$\text{No. ILI cases from Dec in year } y \text{ to Mar in year } y+1 \text{ in Wuhan}$$

$$= \sum \frac{\text{No. ILI}_{\text{Sur}_{da}} \text{ in period 1}}{\text{No. Outp}_{\text{Sur}_d} \text{ in year } y} * (\text{No. Outp}_{\text{HB}_d} \text{ in year } y * \text{P}_{\text{WH vs.HB}})$$

$$+ \sum \frac{\text{No. ILI\_Sur}_{da} \text{ in period 2}}{\text{No. Outp\_Sur}_d \text{ in year } y+1} * (\text{No. Outp\_HB}_d \text{ in year } y+1 * \text{P}_{\text{WH vs.HB}})$$

Where, $No.\text{ILI\_Sur}_{da}$ denotes the number of ILI cases in age group a in sentinel departments of Wuhan hospitals; a is for age categories 0-4, 5-14, 15-24, 25-59, and ≥60 years; d includes internal medicine department, pediatric department, and fever clinic; period 1 denotes Dec 1-31 in year y, period 2 denotes Jan 1 to Mar 31 in year y+1; $No.\text{Outp\_Sur}_d$ denotes the total number of outpatients visiting Wuhan sentinel departments; $No.\text{Outp\_HB}_d$ denotes the total number of outpatient in departments d of all hospitals of Hubei province; $P_{\text{WH vs. HB}}$ denotes the proportion of Hubei province consultations that are specific to Wuhan.

Data for $No.\text{ILI\_Sur}_{da}$ and $No.\text{Outp\_Sur}_d$ was obtained from National ILI surveillance network in Wuhan and Southern China during 2010-2016.[42] We assumed that baseline non COVID-19 ILIs in Wuhan could be well approximated by data from Southern China as the proportion of ILI patients among all consultations was highly correlated between the two locations.[42] The proportion of ILI patients among all

consultations in Southern China during 2019-2020 was higher than that in previous seven seasons. In Southern China, the average proportion of ILI patients in 2019-20 was 1.75 times than that in 2015-16 season.[42] Accordingly, we multiplied the ratio $\frac{No.ILI_{Sur_{da}}}{No.Outp_{Sur_d}}$ for the 2015-16 season by 1.75-fold to estimate the number of ILI cases in Wuhan in 2019-20.

With regards to $No.\text{Outp\_HB}_d$, a linear regression model was used to predict the corresponding consultations in the departments of internal medicine and paediatrics in 2019-2020 in Hubei based on consultations in 2012-2017 because the number of outpatient visits in 2012-2017 showed a clear increase at a linear rate (Fig. S4; adjusted R-square of linear model~ 0.94). This increasing trend could be explained by a gradual increase in overall population size and access to healthcare (i.e., number of hospitals) in Wuhan (Fig. S5). The yearly number of consultations at fever clinics in Hubei were not available from publicly data source. National ILI surveillance shows that the age profile of patients in fever clinics is comparable to that in internal medicine department in Hubei. We assumed the ratio of number of outpatients in fever clinics to be the same as that of internal medicine department in sentinel hospitals during 2012-2016, and could represent the ratio in all hospitals in Hubei. And hence, this ratio was used as a multiplier to estimate the yearly number of consultations at fever clinics in Hubei. The median ratio between 2012-2016 was used as a proxy for 2017-2020. (Table S9)

The annual number of outpatients visiting internal medicine and pediatric departments, and fever clinics was unavailable from publicly data source in Wuhan. And hence we adjusted the $No.\mathrm{Outp\_HB}_d$ using the proportion of total medical visits in Hubei Province occurring in Wuhan as a multiplier to estimate the annual number of consultations in these departments in Wuhan (Table S9).

The age groups differed between estimated ILI cases (i.e., ≤4, 5-14, 15-24, 25-59, and ≥60 years) and reported COVID-19 cases in Wuhan (i.e., ≤19, 20-39, 40-59 and ≥60 years). To break down the above number of ILI cases into the same age groups as that of reported COVID-19 cases, we assumed the age profile of reported ILI cases was consistent with that of these ILI cases for whom respiratory specimens were collected for influenza virus testing in Hubei. The estimated age-stratified number of ILI cases in Wuhan during Dec. 2019 and Mar. 2020 is shown in tables S10.

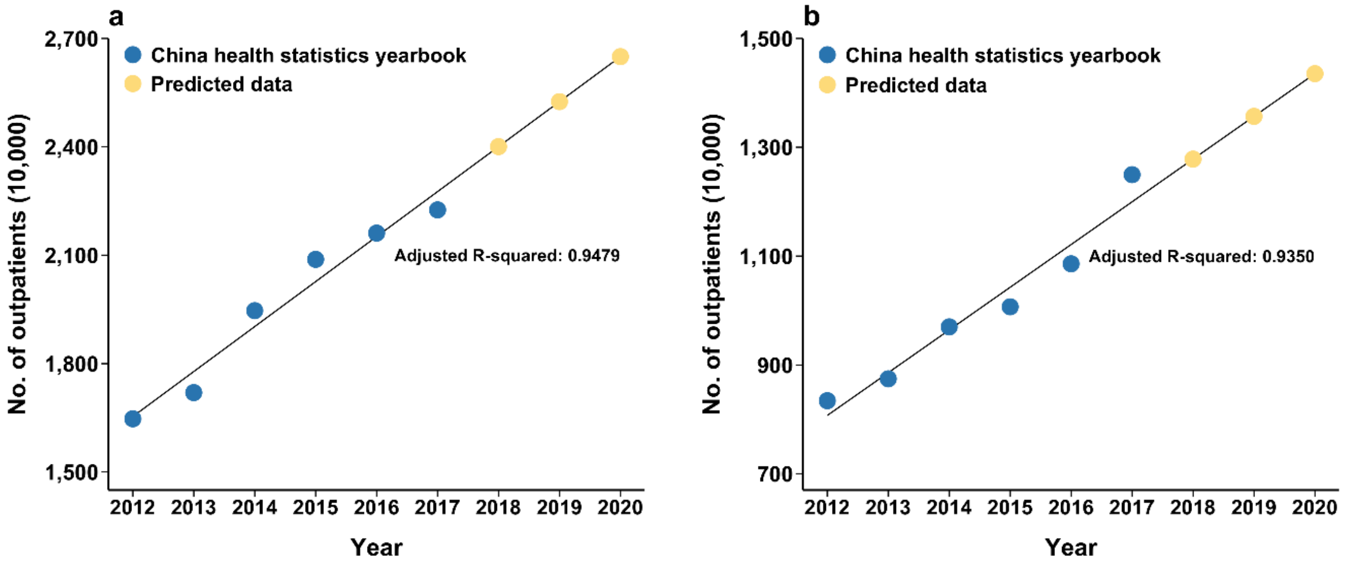

Fig. S4. Number of outpatients at internal medicine departments and paediatric department in Hubei, 2012-2020

A: internal medicine department; B: paediatric department.

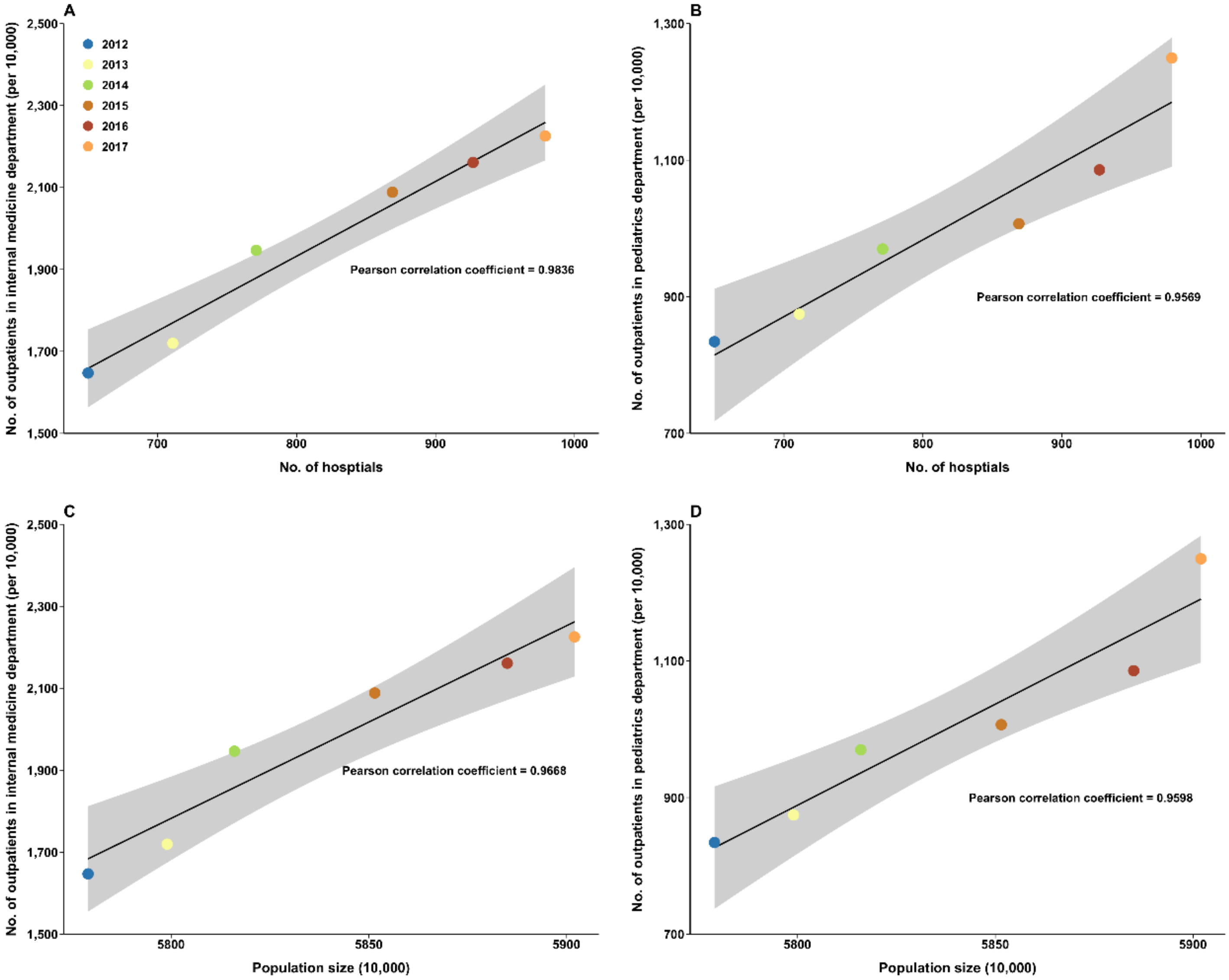

Fig. S5. Correlation of number of outpatients at internal medicine departments and paediatric department separately with population size and number of hospitals in Hubei, 2012-2017

A: correlation of number of outpatients at internal medicine department with number of hospitals; B: correlation of number of outpatients at paediatric department with number of hospitals; C: correlation of number of outpatients at internal medicine department with population size; D: correlation of number of outpatients at paediatric department with population size. Black lines denote the fitted line, and grey shadows denote the lower and upper limits of 95%CI, which were calculated on the basis of Binomial distributions.

Table S9. Estimated number of outpatients in pediatric and internal medicine departments, and fever clinics, Wuhan, 2012-2020

Year	No. outpatients in departments of internal medicine in Hubei (10,000) (a)[ξ]	No. outpatients in departments of pediatrics in Hubei (10,000) (b)[ξ]	The proportion of consultations of fever department among that in department of internal medicine in sentinel hospital (c)[†]	No. outpatients visiting the fever department in Hubei (10,000) (d) = (a) * (c)	The proportion of total medical visits in Wuhan, among those in Hubei province (e)[§]	No. outpatients in the departments of internal medicine in Wuhan (10,000) (f) = (a) * (e)	No. outpatients in the departments of pediatrics in Wuhan (10,000) (g) = (b) * (e)	No. outpatients in the fever departments in Wuhan (10,000) (h) = (d) * (e)
2012	1,647.2	834.2	2.84%	46.7	35.01%	576.7	292.1	16.4
2013	1,719.7	874.5	3.17%	54.5	34.76%	597.7	304.0	18.9
2014	1,946.6	969.9	3.99%	77.7	34.32%	668.0	332.8	26.7
2015	2,088.5	1,006.8	4.13%	86.3	35.64%	744.4	358.9	30.8
2016	2,161.2	1,085.8	3.11%	67.3	37.50%	810.5	407.2	25.2
2017	2,225.5	1,249.6	3.17%	70.6	38.28%	851.8	478.3	27.0
2018	2,400.6	1,278.2	3.17%	76.1	35.33%	848.1	451.6	26.9
2019	2,525.1	1,356.8	3.17%	80.1	35.33%	892.1	479.3	28.3
2020	2,649.6	1,435.3	3.17%	84.0	35.33%	936.0	507.1	29.7

[ξ] Number of outpatients in the department of internal medicine and paediatrics in Hubei during 2012-2017 were obtained from the Chinese Health Statistics Yearbook (Fig S4).[43] A linear regression model was used to estimate the number during 2018-2020.

[†]The ratio of number of outpatients in fever clinics to that in internal medicine during 2012-2016 was obtained from National ILI Surveillance Network in Hubei,[11, 18] and then the median of ratios between 2012-2016 was used for that during 2017-2020.

[§]For 2018-2020, the proportion of total medical visits in Wuhan among those reported for Hubei province was taken as the median of these proportions between 2012-2017.

Table S10. Estimated number of ILI cases in Wuhan, from December 1, 2019 through to March 31, 2020

	Department of Internal medicine		Department of pediatrics		Fever clinics		All
No. ILI cases (a) [ξ]	Dec 1-31, 2015	Jan 1-Mar 31, 2016	Dec 1-31, 2015	Jan 1-Mar 31, 2016	Dec 1-31, 2015	Jan 1-Mar 31, 2016	Dec 1, 2015-Mar 31, 2016
0-19 years	412	1,308	4,489	10,066	19	8	16,302
20-39 years	72	279	781	2,147	3	2	3,284
40-59 years	44	143	477	1,104	2	1	1,771
≥60 years	24	78	259	603	1	0	965
Total	552	1,808	6,006	13,920	25	11	22,322
No. consultations (b) [ξ]	781,638	482,277	981,425	727,110	30,602	205	3,003,257
No. ILI/Consultations (c) = (a) / (b)	Dec 1-31, 2015	Jan 1-Mar 31, 2016	Dec 1-31, 2015	Jan 1-Mar 31, 2016	Dec 1-31, 2015	Jan 1-Mar 31, 2016	Dec 1, 2015-Mar 31, 2016
0-19 years	0.05%	0.27%	0.46%	1.38%	0.06%	3.88%	0.54%
20-39 years	0.01%	0.06%	0.08%	0.30%	0.01%	0.83%	0.11%
40-59 years	0.01%	0.03%	0.05%	0.15%	0.01%	0.43%	0.06%
≥60 years	0.00%	0.02%	0.03%	0.08%	0.00%	0.23%	0.03%
Total	0.07%	0.37%	0.61%	1.91%	0.08%	5.37%	0.74%
Estimated ILI/Consultations (d) = (c)*1.75[†]	Dec 1-31, 2019	Jan 1-Mar 31, 2020	Dec 1-31, 2019	Jan 1-Mar 31, 2020	Dec 1-31, 2019	Jan 1-Mar 31, 2020	Dec 1, 2019-Mar 31, 2020
0-19 years	0.09%	0.47%	0.80%	2.42%	0.11%	6.79%	0.95%
20-39 years	0.02%	0.10%	0.14%	0.52%	0.02%	1.45%	0.19%
40-59 years	0.01%	0.05%	0.09%	0.27%	0.01%	0.74%	0.10%
≥60 years	0.01%	0.03%	0.05%	0.15%	0.01%	0.41%	0.06%
Total	0.12%	0.66%	1.07%	3.35%	0.14%	9.39%	1.30%
Estimated No. outpatients in Wuhan in 2019-2020 (10,000) (e)	892.1	936.0	479.3	507.1	28.3	29.7	2872.5
Estimated No. ILI cases	Dec 1-31, 2019	Jan 1-Mar 31, 2020	Dec 1-31, 2019	Jan 1-Mar 31, 2020	Dec 1-31, 2019	Jan 1-Mar 31, 2020	Dec 1, 2019-Mar 31, 2020

(f) = (d)*(e)							
0-19 years	8,225	44,430	38,368	122,855	314	20,168	234,360
20-39 years	1,432	9,477	6,678	26,206	55	4,302	48,150
40-59 years	874	4,874	4,078	13,476	33	2,212	25,547
≥60 years	475	2,660	2,215	7,354	18	1,207	13,929
Total	11,006	61,441	51,339	169,891	420	27,889	321,986

ξ Obtained from National ILI Surveillance Network in Hubei.[11, 18]

† The average proportion of ILI patients in the 2019-20 season was 1.75 times than that in 2015-16 season. Accordingly, we multiplied (c) in the 2015-16 season by 1.75-fold to estimate the number of ILI cases in Wuhan in 2019-20.

Supplementary file 13: Estimating the number of SARI/pneumonia hospitalizations in Wuhan

We used SARI surveillance in Jingzhou, Hubei Province in 2011 to estimate the number of SARI hospitalization in Wuhan in 2020.[14] Here we assumed that i) the SARI hospitalization rate in Wuhan was same as that in Jingzhou; ii) the ratio of Wuhan SARI hospitalization rate in 2020 to that in 2011 was the same with that of national pneumonia hospitalization rate, which was obtained from the China Health Statistic Yearbook.[43] SARI surveillance in Jingzhou shows that the number of SARI patients during Dec. 2010-Mar. 2011 accounted for 34% of patients seen in a year.[14] And thus, we multiplied total pneumonia hospitalizations by 34% as to obtain the number of SARI hospitalization in Wuhan during Dec. 2019 and Mar. 2020.

Based on the number of pneumonia hospitalization in China in 2010-2018, we used the ratio of the health service index in Hubei to extrapolate to the number of pneumonia hospitalization in Wuhan in 2020. Then age-stratified numbers were estimated assuming the same age profile as SARI cases. The health service index was calculated using the social-demographic index (SDI) methodology from the global burden of diseases studies (GBD),[44, 45] wherein an index value was determined for each variable including hospital beds per capita, provincial GDP per capita, number of doctors per capita and number of nurses per capita.

$$I_{varibale,year,location} = V_{year,location} - \min(V)/(\max(V) - \min(V))$$

Where$I_{varibale,year,location}$ refers the index of the variable in a year in a location; location refers to a province or China; $V_{year,location}$ refers to the observed value of the variable in a year in a location; $\min(V)$ and $\max(V)$ represents the minimum and maximum observed value of the variable in whole year ranges and all locations. Then we computed the geometric mean of all indexes in a location to obtain its health service index (Table S11).

Table S11. Health service index

Region	2010	2011	2012	2013	2014	2015	2016	2017	2018
Nation	0.18	0.21	0.25	0.29	0.32	0.36	0.39	0.44	0.48
Beijing	0.58	0.62	0.66	0.69	0.73	0.77	0.83	0.89	0.96
Tianjin	0.34	0.35	0.37	0.40	0.42	0.45	0.48	0.53	0.55
Hebei	0.14	0.17	0.19	0.22	0.24	0.27	0.30	0.34	0.39
Shanxi	0.22	0.25	0.28	0.30	0.31	0.32	0.34	0.38	0.42
Inner Mongolia	0.25	0.29	0.34	0.39	0.42	0.45	0.47	0.49	0.53
Liaoning	0.32	0.35	0.39	0.43	0.45	0.47	0.47	0.51	0.55
Jilin	0.25	0.28	0.31	0.33	0.36	0.39	0.43	0.44	0.50
Heilongjiang	0.22	0.24	0.27	0.29	0.31	0.33	0.36	0.39	0.41
Shanghai	0.42	0.44	0.46	0.49	0.53	0.57	0.62	0.67	0.72
Jiangsu	0.21	0.25	0.32	0.37	0.42	0.46	0.52	0.56	0.62
Zhejiang	0.27	0.31	0.36	0.41	0.45	0.51	0.56	0.62	0.67
Anhui	0.08	0.10	0.14	0.17	0.20	0.22	0.25	0.29	0.33
Fujian	0.15	0.20	0.25	0.29	0.33	0.35	0.37	0.41	0.46
Jiangxi	0.00	0.09	0.13	0.17	0.19	0.21	0.24	0.28	0.31
Shandong	0.22	0.25	0.30	0.37	0.39	0.41	0.44	0.49	0.54
Henan	0.11	0.13	0.17	0.21	0.24	0.28	0.31	0.36	0.41
Hubei	0.16	0.20	0.24	0.29	0.34	0.39	0.43	0.46	0.50
Hunan	0.12	0.15	0.18	0.22	0.26	0.31	0.36	0.40	0.43
Guangdong	0.16	0.20	0.23	0.27	0.30	0.34	0.38	0.42	0.46
Guangxi	0.08	0.11	0.15	0.18	0.21	0.24	0.27	0.29	0.32
Hainan	0.13	0.17	0.19	0.23	0.26	0.30	0.33	0.36	0.41
Chongqing	0.12	0.16	0.20	0.24	0.28	0.33	0.38	0.43	0.49

Sichuan	0.12	0.17	0.22	0.26	0.30	0.32	0.36	0.41	0.45
Guizhou	0.00	0.06	0.11	0.16	0.20	0.25	0.29	0.35	0.40
Yunnan	0.07	0.10	0.13	0.17	0.19	0.22	0.26	0.31	0.35
Tibet	0.03	0.05	0.00	0.08	0.11	0.15	0.18	0.24	0.28
Shaanxi	0.18	0.21	0.26	0.31	0.34	0.37	0.42	0.48	0.53
Gansu	0.08	0.12	0.15	0.18	0.21	0.22	0.24	0.28	0.32
Qinghai	0.17	0.23	0.26	0.31	0.34	0.37	0.39	0.44	0.47
Ningxia	0.21	0.25	0.28	0.33	0.36	0.38	0.43	0.49	0.52
Xinjiang	0.26	0.30	0.33	0.36	0.38	0.39	0.41	0.44	0.46
Proportion (Hubei vs. Nation)	0.89	0.93	0.96	1.00	1.05	1.10	1.09	1.06	1.04

Supplementary file 14: Estimated disease burden of COVID-19 using ILI consultations, and SARI/pneumonia hospitalizations as a reference

Table S12. Estimated number ILI consultations, and SARI/pneumonia hospitalizations between December 1, 2019 through March 31, 2020 in Wuhan, China

Age (years)	Estimated number of ILI cases	Estimated number of SARI hospitalizations	Estimated number of pneumonia hospitalizations
0-19	234,360	95,530	13,628
20-39	48,150	1,535	219
40-59	25,547	2,094	115
≥60	13,929	4,329	618
All	321,986	103,488	14,580

References

1. Zhang J., *et al.* Evolving epidemiology and transmission dynamics of novel coronavirus disease 2019 outside Hubei Province in China: a descriptive and modeling study. *Lancet Infect. Dis.* **20**, 793-802 (2020).
2. National Health Commission of China. The diagnosis and treatment scheme of novel coronavirus diseases 2019 (Trial version 7th). http://www.gov.cn/zhengce/zhengceku/2020-03/04/content_5486705.htm (accessed March 3 2020).
3. National Health Commission of China. The diagnosis and treatment scheme of novel coronavirus diseases 2019 (Trial version 5th). http://www.gov.cn/zhengce/zhengceku/2020-02/05/content_5474791.htm (accessed February 25 2020).
4. Chinese Thoracic Society. Guidelines for diagnosis and treatment of community-acquired pneumonia in Chinese adults (2016). *Chin. J. Tuberc. Respir. Dis.* **39**, 253-279 (2016).
5. Chinese Thoracic Society. Guidelines for diagnosis and treatment of hospital-acquired pneumonia and ventilator-associated pneumonia in Chinese adults (2018). *Chin. J. Tuberc. Respir. Dis.* **41**, 255-280 (2018).
6. The State Council of the People's Republic of China. Announcement of Wuhan Headquarters for the Control and Treatment of Novel Pneumonia (No. 7) (In Chinese) http://www.gov.cn/xinwen/2020-01/24/content_5472017.htm

(accessed January 24 2020).

7. China Central Television. Report on results of 3-day screening in each district of Wuhan. (In Chinese). http://news.cctv.com/2020/02/20/ARTIfdQ2kV0eRTE4rgX2Aa3D200220.shtml (accessed February 20 2020).

8. People's Daily Online. 4,243,000 households in Wuhan community were screened, reaching an investigating rate of 99.7%. (In Chinese). http://society.people.com.cn/n1/2020/0213/c1008-31585456.html (accessed February 13 2020).

9. Pan A., *et al.* Association of Public Health Interventions With the Epidemiology of the COVID-19 Outbreak in Wuhan, China. *JAMA* **323**, 1915-1923 (2020).

10. The Novel Coronavirus Pneumonia Emergency Response Epidemiology Team. The Epidemiological Characteristics of an Outbreak of 2019 Novel Coronavirus Diseases (COVID-19) — China, 2020. *China CDC Weekly* **2**, 113-122 (2020).

11. Feng L., *et al.* Burden of influenza-associated outpatient influenza-like illness consultations in China, 2006-2015: A population-based study. *Influenza Other Respir. Viruses* **14**, 162-172 (2020).

12. Fowlkes A., *et al.* Incidence of medically attended influenza during pandemic and post-pandemic seasons through the Influenza Incidence Surveillance

Project, 2009-13. *Lancet Respir. Med.* **3**, 709-718 (2015).

13. Shrestha S. S., *et al.* Estimating the burden of 2009 pandemic influenza A (H1N1) in the United States (April 2009-April 2010). *Clin. Infect. Dis.* **52 Suppl 1**, S75-82 (2011).

14. Yu H., *et al.* The substantial hospitalization burden of influenza in central China: surveillance for severe, acute respiratory infection, and influenza viruses, 2010-2012. *Influenza Other Respir. Viruses* **8**, 53-65 (2014).

15. Palekar R. S., *et al.* Burden of influenza-associated respiratory hospitalizations in the Americas, 2010-2015. *PloS One* **14**, e0221479 (2019).

16. Reed C., *et al.* Estimating influenza disease burden from population-based surveillance data in the United States. *PloS One* **10**, e0118369 (2015).

17. Dawood F. S., *et al.* Estimated global mortality associated with the first 12 months of 2009 pandemic influenza A H1N1 virus circulation: a modelling study. *Lancet Infect. Dis.* **12**, 687-695 (2012).

18. Li L., *et al.* Influenza-associated excess respiratory mortality in China, 2010-15: a population-based study. *Lancet Public Health* **4**, e473-e481 (2019).

19. Iuliano A. D., *et al.* Estimates of global seasonal influenza-associated respiratory mortality: a modelling study. *Lancet.* **391**, 1285-1300 (2018).

20. Frost W. H. The Epidemiology of Influenza. *Public Health Rep.* **34**, 1823-1836 (1919).

21. Mamelund S. E., Haneberg B., Mjaaland S. A Missed Summer Wave of the

1918-1919 Influenza Pandemic: Evidence From Household Surveys in the United States and Norway. *Open Forum Infect. Dis.* **3**, ofw040 (2016).

22. Presanis A. M., *et al.* The severity of pandemic H1N1 influenza in the United States, from April to July 2009: a Bayesian analysis. *PLoS Med.* **6**, e1000207 (2009).

23. Wong J. Y., *et al.* Hospitalization Fatality Risk of Influenza A(H1N1)pdm09: A Systematic Review and Meta-Analysis. *Am. J. Epidemiol.* **182**, 294-301 (2015).

24. Bignami S., Assche A. V. Assessing the burden of COVID-19 in Canada.*medRxiv*. https://www.medrxiv.org/content/10.1101/2020.06.14.20130815v1 (2020).

25. Stokes E. K., *et al.* Coronavirus Disease 2019 Case Surveillance - United States, January 22-May 30, 2020. *MMWR* **69**, 759-765 (2020).

26. Centers for Disease Control and Prevention. Overview of Testing for SARS-CoV-2. https://www.cdc.gov/coronavirus/2019-ncov/hcp/testing-overview.html (accessed July 20 2020).

27. United Nations. World Population Prospects 2019. https://population.un.org/wpp/Download/Standard/CSV/ (accessed July 8 2020).

28. Lewnard J. A., *et al.* Incidence, clinical outcomes, and transmission dynamics of hospitalized 2019 coronavirus disease among 9,596,321 individuals

residing in California and Washington, United States: a prospective cohort study. *BMJ* **369**, m1923 (2020).

29. Emily DeRuy. California rethinks coronavirus testing strategy as cases surge. https://www.google.com/amp/s/www.mercurynews.com/2020/07/14/california-rethinks-coronavirus-testing-strategy-cases-surge/amp/ (accessed July 22 2020).

30. California Department of Public Health. Updated COVID-19 Testing Guidance. https://www.cdph.ca.gov/Programs/CID/DCDC/Pages/COVID-19/Updated-COVID-19-Testing-Guidance.aspx (accessed July 22 2020).

31. Washington State Department of Health. Washington State Testing Strategy for COVID-19. https://www.doh.wa.gov/Portals/1/Documents/1600/coronavirus/COVID-19-TestingExecutiveSummary.pdf (accessed July 22 2020).

32. Garg S., *et al.* Hospitalization Rates and Characteristics of Patients Hospitalized with Laboratory-Confirmed Coronavirus Disease 2019 — COVID-NET, 14 States, March 1–30, 2020. *MMWR* **69**, 458–464 (2020).

33. New York State. COVID-19 Testing. https://coronavirus.health.ny.gov/covid-19-testing (accessed July 21 2020).

34. New York City Department of Health and Mental Hygiene (DOHMH) COVID-19 Response Team. Preliminary Estimate of Excess Mortality During the COVID-19 Outbreak — New York City, March 11–May 2, 2020. *MMWR*

69, 603–605 (2020).

35. Weinberger D. M., *et al.* Estimation of Excess Deaths Associated With the COVID-19 Pandemic in the United States, March to May 2020. *JAMA Intern. Med.*, doi: 10.1001/jamainternmed.2020.3391 (2020).

36. Xiao A., Tong Y., Gao C., Zhu L., Zhang Y., Zhang S. Dynamic Profile of RT-PCR Findings from 301 COVID-19 Patients in Wuhan, China: A Descriptive Study. *J. Clin. Virol.*, 104346 (2020).

37. Hao X., Cheng S., Wu D., Wu T., Lin X., Wang C. Reconstruction of the full transmission dynamics of COVID-19 in Wuhan. *Nature*, (2020).

38. Wuhan Municipal Health Commission. Daily report on epidemic situation of COVID-19 in Wuhan. (In Chinese). http://wjw.wuhan.gov.cn/ztzl_28/fk/tzgg/ (accessed May 8 2020).

39. Wuhan Municipal Health Commission. Wuhan revises figures of confirmed COVID-19 cases, deaths. Xinhua News. (In Chinese). http://wjw.wuhan.gov.cn/ztzl_28/fk/tzgg/202004/t20200430_1198387.shtml (accessed September 4 2020).

40. Wuhan Municipal Health Commission. Q&A about confirmed COVID-19 cases and fatalities in Wuhan. Xinhua News. (In Chinese). http://wjw.wuhan.gov.cn/ztzl_28/fk/tzgg/202004/t20200430_1198388.shtml (accessed September 4 2020).

41. Yang J., *et al.* Health-seeking behaviors of patients with acute respiratory

infections during the outbreak of novel coronavirus disease 2019 in Wuhan, China. *Influenza Other Respir. Viruses*, (2020).

42. Chinese National Influenza Center. Chinese Influenza Weekly Report. http://www.chinaivdc.cn/cnic/en/Surveillance/WeeklyReport/202003/t20200319_214599.htm (accessed March 19 2020).

43. China Health Commission. Chinese Health Statistical Yearbook. China Peking Union Medical College Press. (In Chinese). http://navi.cnki.net/KNavi/YearbookDetail?pcode=CYFD&pykm=YSIFE&bh= (accessed March 11 2020).

44. GBD 2017 Influenza Collaborators. Mortality, morbidity, and hospitalisations due to influenza lower respiratory tract infections, 2017: an analysis for the Global Burden of Disease Study 2017. *Lancet Respir. Med.* **7**, 69-89 (2019).

45. GBD 2015 DALYs and HALE Collaborators. Global, regional, and national disability-adjusted life-years (DALYs) for 315 diseases and injuries and healthy life expectancy (HALE), 1990-2015: a systematic analysis for the Global Burden of Disease Study 2015. *Lancet.* **388**, 1603-1658 (2016).
