# Supplementary material for: Disease burden and clinical severity of the first pandemic wave of COVID-19 in Wuhan, China
Source: Nat Commun. 2020 Oct 27;11:5411. doi: 10.1038/s41467-020-19238-2 (PMC7591855; doi:10.1038/s41467-020-19238-2)
Supplement: Supplementary file 3 — Reporting Summary [file 41467_2020_19238_MOESM3_ESM.pdf]

nature research

Corresponding author(s): Hongjie Yu

Last updated by author(s): Sep 19, 2020

Reporting Summary

Nature Research wishes to improve the reproducibility of the work that we publish. This form provides structure for consistency and transparency in reporting. For further information on Nature Research policies, see our Editorial Policies and the Editorial Policy Checklist.

Please do not complete any field with "not applicable" or n/a. Refer to the help text for what text to use if an item is not relevant to your study.
For final submission: please carefully check your responses for accuracy; you will not be able to make changes later.

Statistics

For all statistical analyses, confirm that the following items are present in the figure legend, table legend, main text, or Methods section.

n/a	Confirmed	
☐	☒	The exact sample size (*n*) for each experimental group/condition, given as a discrete number and unit of measurement
☒	☐	A statement on whether measurements were taken from distinct samples or whether the same sample was measured repeatedly
☒	☐	The statistical test(s) used AND whether they are one- or two-sided<br>*Only common tests should be described solely by name; describe more complex techniques in the Methods section.*
☒	☐	A description of all covariates tested
☐	☒	A description of any assumptions or corrections, such as tests of normality and adjustment for multiple comparisons
☐	☒	A full description of the statistical parameters including central tendency (e.g. means) or other basic estimates (e.g. regression coefficient) AND variation (e.g. standard deviation) or associated estimates of uncertainty (e.g. confidence intervals)
☒	☐	For null hypothesis testing, the test statistic (e.g. *F*, *t*, *r*) with confidence intervals, effect sizes, degrees of freedom and *P* value noted<br>*Give P values as exact values whenever suitable.*
☒	☐	For Bayesian analysis, information on the choice of priors and Markov chain Monte Carlo settings
☒	☐	For hierarchical and complex designs, identification of the appropriate level for tests and full reporting of outcomes
☒	☐	Estimates of effect sizes (e.g. Cohen's *d*, Pearson's *r*), indicating how they were calculated

Our web collection on statistics for biologists contains articles on many of the points above.

Software and code

Policy information about availability of computer code

Data collection	EXCEL was used to collected data.
Data analysis	All analyses were performed in R version 3.6.3. The R code to replicate the analyses is available on GitHub at https://github.com/zychenfd/Wuhan-disease-burden.

For manuscripts utilizing custom algorithms or software that are central to the research but not yet described in published literature, software must be made available to editors and reviewers. We strongly encourage code deposition in a community repository (e.g. GitHub). See the Nature Research guidelines for submitting code & software for further information.

Data

Policy information about availability of data

All manuscripts must include a data availability statement. This statement should provide the following information, where applicable:
- Accession codes, unique identifiers, or web links for publicly available datasets
- A list of figures that have associated raw data
- A description of any restrictions on data availability

All aggregated data analyzed in this study are included in the Article and its Supplementary Information file Table S8. Individual-level data on confirmed COVID-19 cases as of March 8, 2020 are available on GitHub at https://github.com/chaolongwang/SAPHIRE, and health seeking behavior survey data are available on GitHub at https://github.com/zychenfd/Wuhan-disease-burden.

Field-specific reporting

Please select the one below that is the best fit for your research. If you are not sure, read the appropriate sections before making your selection.

- [x] Life sciences
- [ ] Behavioural & social sciences
- [ ] Ecological, evolutionary & environmental sciences

Life sciences study design

All studies must disclose on these points even when the disclosure is negative.

Sample size	All morbidity and mortality data were obtained from published literatures and the Hubei Health Commission, mainly including 50339 COVID-19 confirmed cases and 3869 COVID-19 deaths. The sensitivity of PCR assay were obtained from a published clinical study with a total of 301 COVID-19 cases in Wuhan. We also conducted a health-seeking behavior survey in Wuhan, collecting data from 3411 Wuhan citizens, to estimate the probability of seeking medical care of patients with acute respiratory infections during COVID-19 outbreak, which served as an important parameter for our estimation. This manuscript is accepted by Influenza and Other Respiratory Virus. We summarized and synthesized data from aforementioned multiple sources to produce best estimations of clinical severity and disease burden of COVID-19 in Wuhan.
Data exclusions	One imported COVID-19 case was excluded, because our objective is to quantify the clinical severity and disease burden caused by local intense community transmissions of COVID-19 in Wuhan.
Replication	All aggregated data analyzed in this study are included in the Article and its Supplementary Information files. Individual-level data on confirmed COVID-19 cases as of March 8, 2020 are available on https://github.com/chaolongwang/SAPHIRE, and health seeking behavior survey data are available on GitHub at https://github.com/zychenfd/Wuhan-disease-burden. The R code to replicate the analyses is available on GitHub at https://github.com/zychenfd/Wuhan-disease-burden.
Randomization	Second-hand data were used for our analysis and no randomization was involved. Please refer to the brief summary iin above 'Sample size' section.
Blinding	Second-hand data were used for our analysis and no blinding was involved. Please refer to the brief summary in above 'Sample size' section.

Reporting for specific materials, systems and methods

We require information from authors about some types of materials, experimental systems and methods used in many studies. Here, indicate whether each material, system or method listed is relevant to your study. If you are not sure if a list item applies to your research, read the appropriate section before selecting a response.

Materials & experimental systems

n/a	Involved in the study	
☒	☐	Antibodies
☒	☐	Eukaryotic cell lines
☒	☐	Palaeontology and archaeology
☒	☐	Animals and other organisms
☒	☐	Human research participants
☒	☐	Clinical data
☒	☐	Dual use research of concern

Methods

n/a	Involved in the study	
☒	☐	ChIP-seq
☒	☐	Flow cytometry
☒	☐	MRI-based neuroimaging

This checklist template is licensed under a Creative Commons Attribution 4.0 International License, which permits use, sharing, adaptation, distribution and reproduction in any medium or format, as long as you give appropriate credit to the original author(s) and the source, provide a link to the Creative Commons license, and indicate if changes were made. The images or other third party material in this article are included in the article's Creative Commons license, unless indicated otherwise in a credit line to the material. If material is not included in the article's Creative Commons license and your intended use is not permitted by statutory regulation or exceeds the permitted use, you will need to obtain permission directly from the copyright holder. To view a copy of this license, visit http://creativecommons.org/licenses/by/4.0/
